# Encapsulation of Silicon Nano Powders via Electrospinning as Lithium Ion Battery Anode Materials

**DOI:** 10.3390/ma16093566

**Published:** 2023-05-06

**Authors:** Man Xiong, Xuan Bie, Yawei Dong, Ben Wang, Qunchao Zhang, Xuejun Xie, Tong Liu, Ronghua Huang

**Affiliations:** 1 School of Power & Mechanical Engineering, Wuhan University, Wuhan 430072, China; 2 School of Materials Science and Engineering, Hubei University, Wuhan 430060, China; 3 School of Chemical Engineering and Pharmacy, Wuhan Institute of Technology, Wuhan 430205, China

**Keywords:** silicon anodes, lithium ion battery, electrospinning, encapsulation

## Abstract

Silicon-containing polyester from tetramethoxysilane, ethylene glycol, and o-Phthalic anhydride were used as encapsulating materials for silicon nano powders (SiNP) via electrospinning, with Polyacrylonitrile (PAN) as spinning additives. In the correct quantities, SiNP could be well encapsulated in nano fibers (200–400 nm) using scanning electron microscopy (SEM). The encapsulating materials were then carbonized to a Si-O-C material at 755 °C (Si@C-SiNF-5 and Si@C-SiNF-10, with different SiNP content). Fiber structure and SiNP crystalline structure were reserved even after high-temperature treatment, as SEM and X-ray diffraction (XRD) verified. When used as lithium ion battery (LIB) anode materials, the cycling stability of SiNPs increased after encapsulation. The capacity of SiNPs decreased to ~10 mAh/g within 30 cycles, while those from Si@C-SiNF-5 and Si@C-SiNF-10 remained over 500 mAh/g at the 30th cycle. We also found that adequate SiNP content is necessary for good encapsulation and better cycling stability. In the anode from Si@C-SiNF-10 in which SiNPs were not well encapsulated, fibers were broken and pulverized as SEM confirmed; thus, its cycling stability is poorer than that from Si@C-SiNF-5.

## 1. Introduction

Silicon is regarded as the next generation of lithium ion battery (LIB) anode materials for its high capacity (4200 mAh/g), low delithiation potential (0.2 V), and abundant reserve (the second-highest reserve element in the Earth’s crust, next to oxygen). However, the volume expansion after lithium insertion leads to anode pulverization and breakage, thus greatly decreasing its capacity. This prevents the commercial application of silicon anodes [1,2,3].

Many methods are verified to be effective in preventing breakage of silicon anodes during the lithiation/delithiation procedure. The most frequently used methods include nanometer sizing of silicon and encapsulation of silicon with a carbon shell. Nanometer sizing of silicon down to 150 nm will relieve the stress caused by the volume expansion [4,5].

Nano clusters, nano powders, nano films, nanowires, and nano tubes, etc., can all assist in providing higher capacity after long cycles [6,7,8,9,10,11,12,13,14,15]. However, nano-sized materials are hard to produce in a scalable manner, such as chemical vapor phase precipitation and plasma evaporation and condensation, and usually bring the problem of agglomeration and irreversible capacity due to their high specific area and more surface defects which are active in forming Li-Si compounds [16]. However, the pulverization issue is not minimized enough to avoid volume changes and continuous SEI buildup. Other structures, especially an encapsulated porous structure, have been confirmed to improve anode cycling stability due to the presence of interior void spaces buffering the changes in volume and stabilizing solid electrolyte interface (SEI) formation [17,18,19,20].

The encapsulation of silicon anodes with carbon shells has also been studied. Outer carbon shells not only increase electrical conductivity, which makes lithiation/delithiation easier, but also encapsulate silicon materials in a porous, relatively confined space that prevents the silicon anodes from pulverization. Further, silicon materials are also isolated from the electrolytes. Electrolytes usually generate some compounds with silicon materials via electrochemical reactions, thus leading to irreversible capacity. Many encapsulation methods have been reported, such as ball milling, spray drying, etc. Ball milling is a facile physical method, but it decreases the crystalline degree of silicons and carbons, and generates many defects in the surface, which leads to side electrochemical reactions when the lithium ion is inserted. Spray drying can produce porous carbon-encapsulated silicon powders within a short time. However, it cannot completely form an outer shell, and only flexible graphene can be directly applied.

Here, we used electrospinning to form a carbon outer shell for silicon nano powders (SiNP). As suggested, the matrices for silicon anodes are also important for obtaining stable LIB storage [2]. Carbon fibers seem to be an effective matrix for improving the electrochemical performance of silicon anode materials [21]. Electrospinning is a spinning method under higher electric potential. The electric field provides an electric charged surface for the injected solution, which accelerates the evaporation of the solvent. Additionally, the high electric potential elongates the solution drops and orients them to the collector. Under the electric field, electrospinning can easily form slim fibers or micro powders with high porosity. Further, this technology makes it easy to fabricate hybrid materials simply by modification of the components of the solution. Many lithium-ion-battery-related materials, including anodes, cathodes, separators, etc., can be obtained using this technology [22,23].

**Figure 1:**
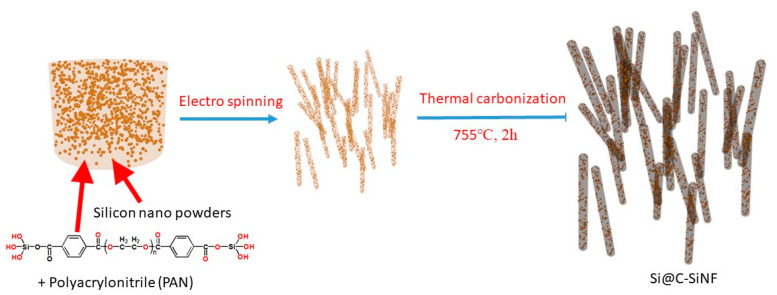
Schematic illustration of the synthesization process of the SiNPs (SiNP) encapsulated in fibers via electrospinning followed by thermal carbonization.

This research was based on SiNPs. A laboratory-synthesized polyester containing silicon (Si-PET) was used as an encapsulant, and Polyacrylonitrile (PAN) was used as the spinning additive. The fibers were then thermally carbonized under an argon atmosphere at 755 °C for 120 min (Figure 1). The thermal carbonization of Si-PET generated a porous structure according to our research [24,25]. The encapsulation of SiNPs were identified using scanning electron microscopy (SEM), and X-ray diffraction (XRD) and thermogravimetric analysis (TA) were also adopted to characterize the materials. As lithium ion battery anode materials, their electric performance was then evaluated.

## 2. Experimental Section

### 2.1. Materials

SiNPs were commercial products from Shanghai CW Nano Tech. CO., Shanghai, China (CW-Si-001, spheres with 30 nm dimeter, 42.4 m^2^/g specific area, and 99.9% purity). Silicon-containing polyester (Si-PET) was laboratory-synthesized from Tetramethoxysilane, ethylene glycol, and o-Phthalic anhydride according to [24,25]. PAN and the solvent for electrospinning, N, N-dimethyl formamide (DMF), were commercially obtained at analytical grade from Sinopharm Chemical Reagent Co., Ltd., Beijin, China. Other reagents for the fabrication of cells, including lithium cathodes, polyvinylidene fluoride (PVDF) binders, LiPF6 electrolyte, N-methyl-2-pyrrolidone (NMP), Cu coil, ethylene carbonate (EC), diethyl carbonate (DEC), and acetylene black (super-*p*) were all commercial products at analytical grade.

### 2.2. Methods

The surface morphology was observed using scanning electron microscopy (SEM, MIRA3, TESCAN, Brno, Czech Republic) with 200~30 kV as the accelerating voltage after coating with gold. X-ray diffractions were recorded on a D8 Advance (Bruker AXS) by using a Cu Ka (λ = 1.5405Å) at 40 mA, 40 kV with 2θ range 10–80°. Thermogravimetric analysis (Differential Scanning Calorimetry (DSC), thermal gravity (TG), and differentiate thermal gravity (DTG)) was tested using a Henven HTG-1 analyzer at a heating rate of 10 °C min^−1^ from room temperature to 755 °C in argon. Fourier transform infrared spectroscopy (FTIR, Thermo 5700, Thermo Fisher, China) was performed from 400 to 4000 cm^−1^. Electrospinning was carried out using a 1 SS-X3 electrospinning instrument (Ucalery, Beijing, China).

### 2.3. Fabrication of Nano Silicon-Containing Fibers

A total of 0.20 g PAN was dissolved in 1.80 g DMF at 60 °C by stirring, then 1.0 g Si-PET and 0.20 g hexadecyl trimethyl ammonium bromide were added and stirring resumed until a clear solution was obtained. Then, the determined weight of SiNPs (0.0, 0.05 and 0.10 g, respectively) was added and stirred to form homogeneous mixtures. The temperature was kept at 60 °C during the whole procedure.

Then, the above mixtures were placed in a syringe and delivered by a programmable pump with a feeding rate of 1 mL/h and temperature at 50 °C. The voltage was variable at a range of 0–20 kV and the distance between the two electrodes was 20 cm.

Finally, the collected fibers were reduced at 755 °C with a heating rate of 2 °C min^−1^ under Ar atmosphere for 2 h. The obtained sample was denoted as Si@C-SiNF-0, Si@C-SiNF-5, or Si@C-SiNF-10 up to the weight of SiNP.

### 2.4. Electrochemical Measurements

Electrochemical characterization was performed with CR2032 coin cells (composed of Celgard 2300 film, lithium foils, and the working electrodes) at room temperature. The working electrodes were prepared by mixing active material, super *p*, and PVDF (which is dispersed in NMP) in a weight ratio of 8:1:1. The mixture was ground into homogeneous slurry with a Mini-mill (Fritsch, pulverisette 23, Idar-Oberstein, Germany) for 15 min. Then, the slurry was coated on a clean Cu foil and dried in a vacuum oven at 65 °C for 24 h. The average mass content of electrode was 1.5 mg·cm^−2^. All the cells were assembled in an argon-filled glove box with water/oxygen content lower than 0.1 ppm. A total of 1 mol·L^−1^ LiPF6 dissolved in ethylene carbonate/diethyl carbonate (EC/DEC, 1:1 by vol.) was used as the electrolyte.

Charge/discharge measurements were performed by a LAND (CT2001A) battery-test system in the voltage range of 0.01–2.0 V. Cyclic voltammetry curves (CV) were measured at a scanning rate of 0.1 mV s^−1^ within the potential range of 0.01–2.0 V vs. Li/Li^+^ using an electrochemistry working station (Squidstat Plus). The electrochemical impedance spectroscopy (EIS) test was carried out in the frequency range from 100 kHz to 10 mHz on an electrochemical workstation (Squidstat Plus).

## 3. Results and Discussion

### 3.1. Micrograph Structure of Fibers

**Figure 2:**
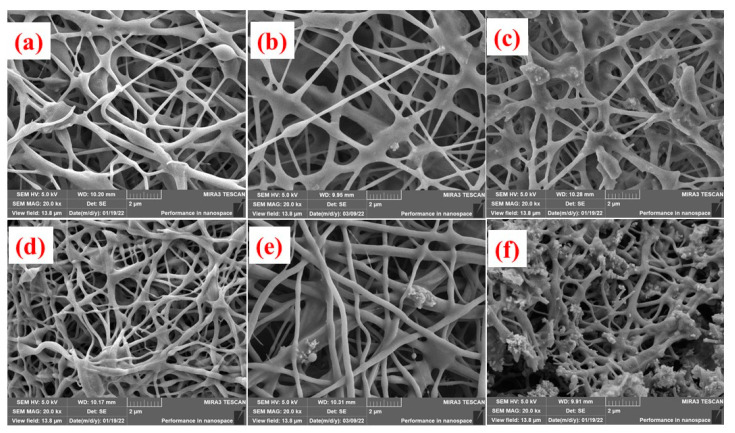
SEM of fibers with different silicon nano powder (SiNP) contents. (**a**–**c**) are those before thermally carbonization, with 0.0%, 5.0%, and 10.0% SiNPs, respectively, while (**d**–**f**) are those that have been carbonized, from Si@C-SiNF-0, Si@C-SiNF-5, and Si@C-SiNF-10. All graphs were taken with ×20.0 K magnification.

Figure 2 presents the SEM of silicon-containing fibers and their precursors before carbonization. The fibers can be clearly observed with diameters of about 400 nm. Further, when with SiNPs, a smooth fiber surface was found, as in Figure 2a. Additionally, if 5.0% SiNPs were added, still no SiNPs could be found, as in Figure 2b. This means that the SiNPs were perfectly encapsulated in fibers. As SiNP content increased to 10.0%, some SiNPs obviously percolated out of fibers, meaning that they were not well encapsulated, as in Figure 2c. Carbonization leads to volume shrinkage according to our experiences, which might be negative for encapsulation. After carbonization, the surface of Si@C-SiNF-0 still remained smooth, as in Figure 2d. For Si@C-SiNF-5, more SiNPs were found, as shown in Figure 2e, but most of the SiNPs were encapsulated. However, for Si@C-SiNF-10, SiNPs percolated out of fibers more seriously, implying complete encapsulation as shown in Figure 2f. From the above, it can be concluded that electrospinning, followed by carbonization at 755 °C, can form encapsulation for s, at a content suitable for SiNPs.

### 3.2. Thermal Analysis of Fibers

**Figure 3:**
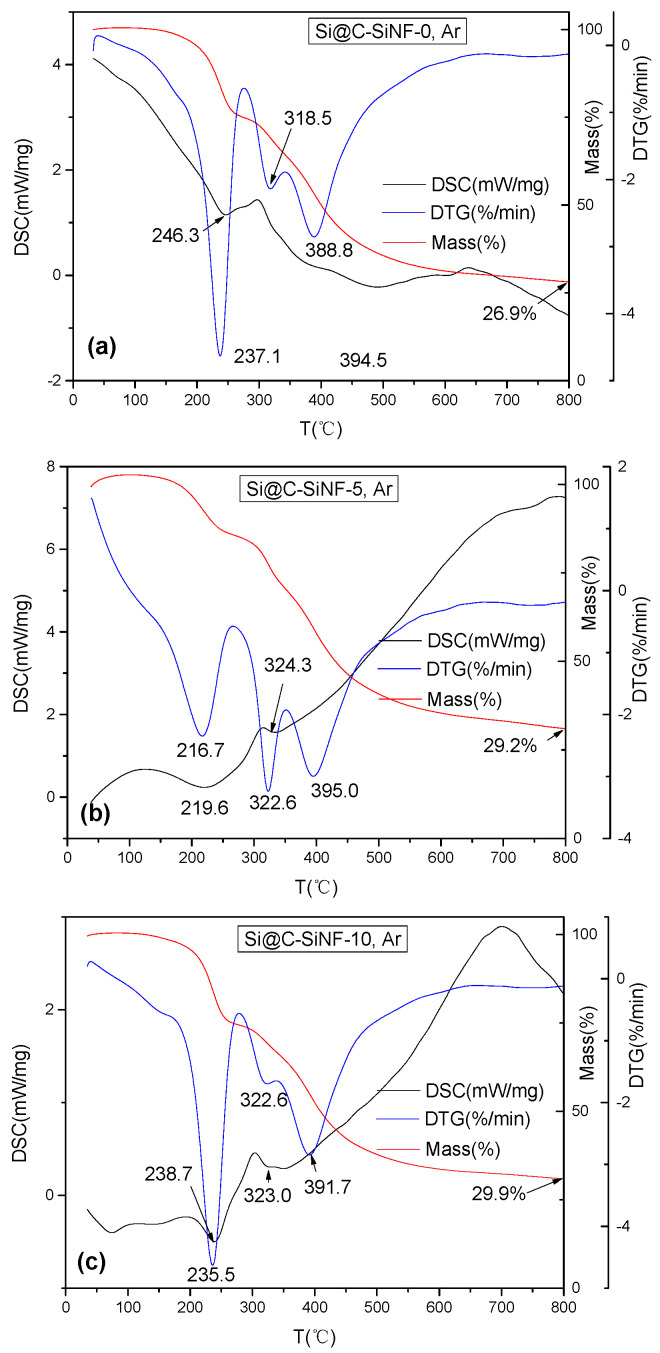
Thermogravimetric analysis (Differential Scanning Calorimetry (DSC), thermal gravity (TG), and differentiate thermal gravity (DTG)) of precursors for (**a**) Si@C-SiNF-0, (**b**) Si@C-SiNF-5, and (**c**) Si@C-SiNF-10 under Ar atmosphere.

Figure 3 presents the thermal analysis fibers with and without SiNPs. All three samples showed three mass loss peaks, accompanied by three exothermal peaks, at similar temperature ranges (200–250 °C, 300–350 °C, and 350–400 °C). This means that no phase transition occurred, but there was degradation of fibers. According to our experiences, silicon-containing polyesters synthesized by our group give mass loss peaks at 200–250 °C and 350–400 °C. Ordinarily, commercial polyethylene terephthalates are pyrolyzed at 377–527 °C [26], which is implied by the peaks at 350–400 °C. The mass loss peaks at 200–250 °C should be assigned to the end-group degradation. The commercial polyethylene terephthalates contain high molecular and regularly arranged chains. However, those prepared in this research are not well structured, containing lower molecular weights and more end groups, such as -OCH_2_CH_2_O- and -COOH. The activation energy for these groups’ degradation was lower, thus showing a mass loss peak at lower temperature. The mass loss peaks at 300–350 °C can be assigned to the addition of PAN. The thermal decomposition PAN generally induces chemical reactions, such as cyclization (up to 250 °C), with very small mass loss, degradation (up to about 300 °C) with obvious mass loss and thermal crosslinking fragmentation (above 400 °C) [27,28]. Therefore, mass loss peaks at 200–250 °C and 350–400 °C might also partly result from PAN, but the peaks at 300–350 °C should be only assigned to it.

The influences of s could be also verified. The peak temperature and A.U. were different, but hard to explain. The most obvious explanation is that the residue weights increase. Even if they are not linearly dependent on silicon content, the residue weight of Si@C-SiNF-0 is 26.9%, and those of Si@C-SiNF-5 and Si@C-SiNF-10 are 29.6% and 29.9%, respectively.

### 3.3. X-ray Diffraction of Fibers

**Figure 4:**
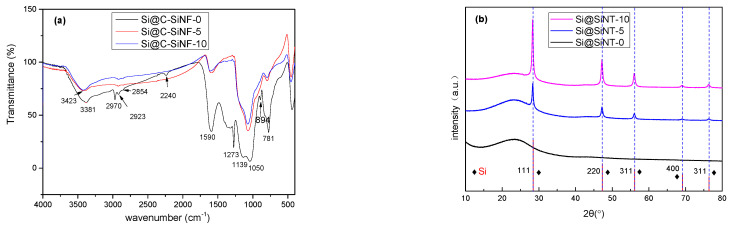
Fourier Transform Infrared Spectrometer (FT IR) (**a**) and X-ray diffraction (XRD) (**b**) of Si@C-SiNF-0, Si@C-SiNF-5, and Si@C-SiNF-10.

Figure 4a presents the FT IR spectra of Si@C-SiNF-0, Si@C-SiNF-5, and Si@C-SiNF-10. The stretching vibration peak of C-H at 2830–2990 cm^−1^, the stretching vibration peak of C = C at 1590 cm^−1^, and the C-N and C-O vibration peak in 1358–985 cm^−1^ can be observed. It is noted that the 2242 cm^−1^ of the C≡N absorption peak, indicating the insufficient cyclization of PAN, can be observed for Si@C-SiNF-0, but not for the other two samples [28]. Si@C-SiNF-0 presents strong absorption of C-H (2970 and 1273 cm^−1^, as well as a weak absorption at 894 cm^−1^), indicating that SiNPs additionally affected the carbonization of PAN. Further, the absorption bands at 1050 (Si-O-Si), 1139 (Si-O-C or C-O-C), and 781 (Si-O-Si) cm^−1^ prove the existence of Si-O-C or Si-O-Si units [24].

In Figure 4b, XRD peaks are identified according to Joint Committee on Powder Diffraction Standards (JCPDS). The 2θ broad peak of silicon dioxide (resulting from the silicon-containing polyesters) or carbon from 20° to 25° can be observed for all samples [29,30,31]. The typical 2θ peaks of silicon crystalline at 28.4°, 47.4°, 56.2°, 69.2°, and 76.5° (for (111), (220), (311), (400), and (311), based on standard ICCD pdf card 92-0092) are clearly identified, dependent on the feeding ratio [32]. Si@C-SiNF-5 gives peaks lower than Si@C-SiNF-10. Further, the precursors of this research contained not only silicon, but also carbon, which might generate SiC at high temperatures. In Figure 4b, some usual 2θ peaks of SiC (35.7°, 41.4°, 60.0°, 71.8°, and 75.4° for (111), (200), (220), (311), and (222), etc., respectively) cannot be observed [33]. Thus, it can be concluded that electrospinning, followed by thermal carbonization at 755 °C, perfectly provides encapsulation without the generation of SiC, which would decrease the capacity if used as anodes.

### 3.4. Electrochemical Performance of Fibers as Anodes and Influences

**Figure 5:**
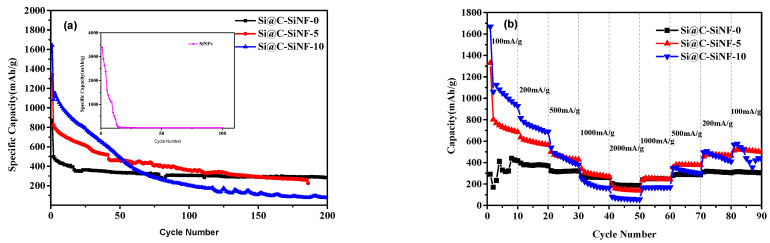
Cycling stability of anodes from fibers with different content of s at 100 mA/g (**a**) and their rate capability at various current densities (100–2000 mA/g) (**b**). In (**a**), the inner cycles are those from pure SiNPs, which are quickly broken.

Cycling stability and rate capability of anodes from Si@C-SiNFs are presented in Figure 5. The anode from pure s (inner figure in Figure 5a) is broken quickly as the capability decreases to ~10 mAh/g within 30 cycles, while those from Si@C-SiNF-5 and Si@C-SiNF-10 keeps over 500 mAh/g at the 30th cycle. This strongly confirms that the encapsulation can increases the cycling stability of anodes. Compared to anodes without s (Si@C-SiNF-0), Si@C-SiNF-5 and Si@C-SiNF-10 show higher initial capacities but lower cycling stability. Additionally, this trend is more obvious if SiNP content increases. Figure 5b confirms that the higher SiNP content is negative for rate capability. Even giving very low capacities at low current density (100 mAh/g), the anode from Si@C-SiNF-0 presents capacities greatly higher than those from Si@C-SiNF-5, and then Si@C-SiNF-10, at high current density (especially at 2000 mA/g).

**Figure 6:**
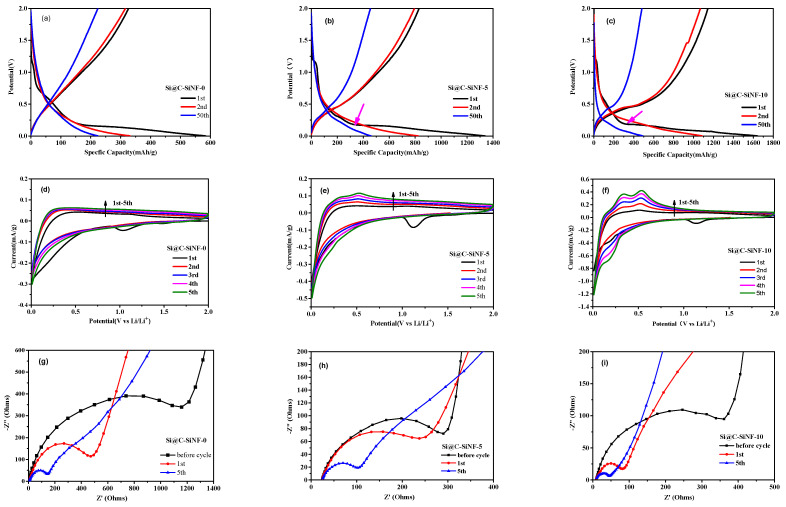
Electrochemical analysis for Si@C-SiNF-0, Si@C-SiNF-5, and Si@C-SiNF-10. (**a**–**c**) Discharge–charge curves cycled between 0.01 and 2.0 V under a current density of 200 mA g^−1^ for the 1st, 2nd, and 50th cycles. (**d**–**f**) Cyclic voltammetry curves measured in the voltage range of 0.01–2.0 V with a scan rate of 0.1 mV s^−1^ and (**g**–**i**) Nyquist diagrams.

Figure 6 presents the discharge–charge curves, cyclic voltammetry curves, and Nyquist diagrams for anodes from Si@C-SiNFs. The discharge–charge curves of Si@C-SiNF-5 and Si@C-SiNF-10 show an obvious discharge plateau of silicon anodes at the first cycle (marked by blue arrows, Figure 6b,c). Si@C-SiNF-0 does not contain SiNPs (Figure 6a). The first Coulombic efficiency of Si@C-SiNF-0, Si@C-SiNF-5, and Si@C-SiNF-10 increases from 55.8%, to 61.91% and 69.63% because the crystalline structure of SiNPs induces fewer irreversible electrochemical reactions.

Cyclic voltammetry curves (Figure 6d–f) of all three samples show reductive peaks at ~1.2 V only at the first cycle, corresponding to the SEI film formation [34]. One cathodic peak at ~0.01 V (lithiation of crystal Si) and two anodic peaks at 0.32 and 0.51 V (delithiation of Li-Si phases, accompanied by formation of amorphous Si), can be clearly observed after subsequent cycles, with increasing intensities, for the activation of silicon crystals [35,36]. The cathodic peak at ~0.2 V at subsequent cycles (lithiation of amorphous Si) can be observed only for Si@C-SiNF-10 after the first cycle, and also show increasing intensities during the charge/discharge procedure. Obviously, these peak intensities are also dependent on SiNP content.

The Nyquist plots of anodes are presented in Figure 6g–i. All Nyquist plots present a semicircle in the high-frequency region and a straight line in the low-frequency region. The former corresponds to charge transfer resistance (Rct) and/or resistance from the SEI layer (R_SEI_), while the latter reflects Li^+^ diffusion resistance in solid electrode [37,38]. The charge transfer resistance (Rct) of Si@C-SiNF-0 before cycling is much larger than that of Si@C-SiNF-5 and Si@C-SiNF-10. The carbonization of silicon-containing polyesters in this study led to a Si-C-O mixture, not pure carbons with electric resistance is higher than the monoatomic silicon. Therefore, the SiNPs in Si@C-SiNF-5 and Si@C-SiNF-10 showed decreased resistance. Further, Rct decreased after the first and fifth cycles. Usually, Rct would increase after cycling for the formation of SEI film [39]. Additionally, less variation of Rct means the stability of SEI films [40]. Chen et al. explained that the decrease of Rct is due to the slow wetting of an electrolyte into porous electrodes and the increased electrical conductivity of the silicon during the charge–discharge process [41]. In case of the straight lines in the low-frequency region, Si@C-SiNF-0 and Si@C-SiNF-5 show decreased slopes as cycling continues, which means that Li^+^ diffusion resistance decreased for the same reason as Rct. However, for Si@C-SiNF-10, after the fifth its slope cycle increases, indicating increasing Li^+^ diffusion resistance. The lithiation/delithiation procedure usually generates a porous structure that facilitates the Li^+^ diffusion resistance, and thus this result is hard to understand.

**Figure 7:**
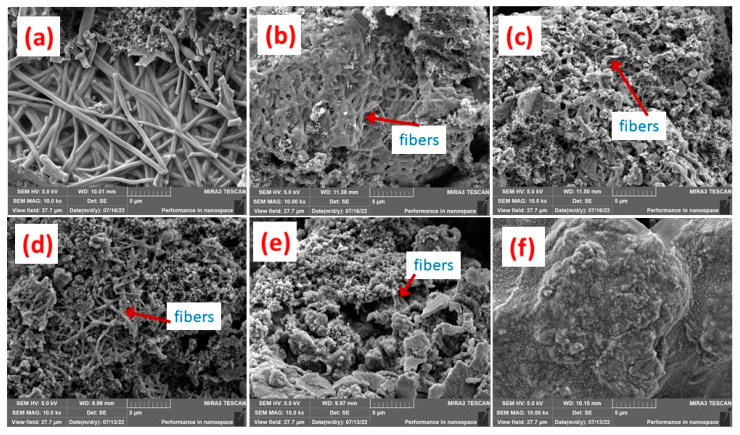
Scanning electron microscopy (SEM) images of anodes from Si@C-SiNFs with different SiNPs, in which (**a**–**c**) are from Si@C-SiNF-0, Si@C-SiNF-5, and Si@C-SiNF-10, respectively, before cycling; while (**d**–**f**) are correspondingly from those after the 100th cycle at 100 mA h g^−1^.

Scanning electron microscopy (SEM) images were adopted to check the integrity of the anodes from Si@C-SiNFs before and after cycling (Figure 7). Figure 7a–c are those before cycling. It can be found that anodes from Si@C-SiNF-0 and Si@C-SiNF-5 present clear fibers, while those from Si@C-SiNF-10 present relatively shorter ones. The fabrication of the cell includes a ball-milling procedure. Si@C-SiNF-10 contains higher content of SiNPs, which makes the fibers crisper and easier to be break during ball-milling. After the 100th cycle, cells were disassembled, and electrodes without further treatment, were SEM photographed. It can be found that the anode from Si@C-SiNF-0 still remained integral and the fibers kept their original shape, except for some white spheres doped on the surface as shown in Figure 7d. For Si@C-SiNF-5, the anode was not pulverized, but became porous, and fibers could still can be found with difficulty, as shown in Figure 7e. Conversely, on the surface of the anode from Si@C-SiNF-10, no fibers or porous structures were observed, implying a broken and pulverized anode. To maintain cycling stability, the integrity of the SEI film is necessary [42]. The pulverization might result from the poorer mechanical properties mentioned before, as well as the more drastic volume variation during the lithiation/delithiation procedure (resulting from its high content of SiNPs). This could be the reason for its lower cycling stability and higher Li^+^ diffusion resistance of the anode from Si@C-SiNF-10.

## 4. Conclusions

Silicon nano powders (SiNPs) were successfully encapsulated in fibers via a electrospinning–carbonization procedure with the correct levels. Powders kept their crystalline structure even after high-temperature carbonization of the encapsulating materials. When used as lithium ion battery anode materials, the encapsulation of SiNPs in fibers can increase their cycling stability compared to the untreated. Additionally, the correct SiNP content is also necessary. Higher content of SiNPs led to incomplete encapsulation, and more drastic volume variation, and thus led to the easy breaking of anodes.

## Data Availability

No new data were created or analyzed in this study. Data sharing is not applicable to this article.
